# Working Memory Alterations Plays an Essential Role in Developing Global Neuropsychological Impairment in Duchenne Muscular Dystrophy

**DOI:** 10.3389/fpsyg.2020.613242

**Published:** 2021-01-15

**Authors:** Rahul Tyagi, Harshita Arvind, Manoj Goyal, Akshay Anand, Manju Mohanty

**Affiliations:** ^1^Neuroscience Research Lab, Department of Neurology, Postgraduate Institute of Medical Education and Research, Chandigarh, India; ^2^Department of Neurology, Postgraduate Institute of Medical Education and Research, Chandigarh, India; ^3^Department of Neurosurgery, Postgraduate Institute of Medical Education and Research, Chandigarh, India

**Keywords:** neuropsychology, DMD, Duchenne muscular dystrophy, intelligence, working memory

## Abstract

**Background:**

Neuropsychological profile of Indian Duchenne muscular dystrophy (DMD) subjects remains unidentified and needs to be evaluated.

**Methods:**

A total of 69 DMD and 66 controls were subjected to detailed intelligence and neuropsychological assessment. The factor indexes were derived from various components of Malin’s Intelligence Scale for Indian Children (MISIC) and Rey Auditory Verbal Learning Test (RAVLT).

**Results:**

Poor verbal and visual memory profiles were demonstrated by DMDs, which include RAVLT-immediate recall (IR) (*p* = 0.042), RAVLT-delayed recall (DR) (*p* = 0.009), Rey–Osterrieth complex figure test (RCFT)-IR (*p* = 0.001), and RCFT-DR (*p* = 0.001). RAVLT-memory efficiency index demonstrated poor verbal memory efficiency (*p* = 0.008). Significant differences in the functioning of working memory axis [RAVLT T1 (*p* = 0.015), recency T1 (*p* = 0.004), Digit Span Backward (*p* = 0.103)] were observed along with reduced performance in visuomotor coordination, visuospatial, and visual recognition abilities. Block designing efficiency index and attention fraction showed a normal performance in DMD kids.

**Conclusion:**

Working memory deficits were found to be the crucial element of cognitive functioning in DMD cases. Working memory interventions may be beneficial to improve the neuropsychological profile in DMD.

## Introduction

Duchenne muscular dystrophy (DMD) is a rare X-linked inherited progressive neuromuscular dysfunction caused by pathogenic variations in the *DMD* gene encoding a rod-shaped protein called dystrophin, which provides an anchor between the cytoskeleton and extracellular matrix resulting in proper muscular integrity and strength (Koenig et al., 1987; Kunkel et al., 1987). The absence of dystrophin results in loss of structural integrity leading to progressive muscular weakness ultimately resulting in loss of ambulation and early death in the twenties due to respiratory and cardiac dysfunction (Moser, 1984; Mercuri and Muntoni, 2013).

Though *Duchenne de Boulogne* reported intellectual disability in his descriptions (Duchenne, 1868), the cause of impairment of cognitive domains remain undetermined. Several initial reports indicated a moderate reduction in the intelligence quotient (IQ) in approximately one-third of patients with DMD with more severe affection of verbal compared to performance IQ (Cohen et al., 1968; Cotton et al., 2001, 2005). These studies also suggested the crucial role of dystrophin protein in the development of the intellectual trajectory. A comparison of DMD to patients with similar conditions like spinal muscular atrophy provided evidence of the cognitive deficits not due to motor dysfunction (Billard et al., 1992). The presence of non-progressive mental retardation is a prominent clinical feature in DMD (Bresolin et al., 1994). We have also reported non-progressive intellectual and neuropsychological deficits in our DMD cohort (Tyagi et al., 2019b).

Subsequent research reported variable degrees of impairment in specific cognitive domains involving immediate verbal memory, visual memory, and attention (Anderson et al., 1988). Donders and Taneja (2009) reported significant deficits in the delayed verbal recall without any difference in immediate memory tasks in DMD patients compared to their unaffected siblings (Donders and Taneja, 2009; Anand et al., 2015). Recent studies have also reported deficits in the executive functioning including planning and inhibition among DMD patients (Mento et al., 2011) when compared to controls (motor impairment: SMA, typically developing children: rheumatoid arthritis, and unaffected sibling). The brain is also an important site of dystrophin expression, where deficit may cause severe mechanical alterations. Along with full-length dystrophin protein (Dp427), short dystrophin isoforms including Dp260, Dp140, Dp116 Dp71, and Dp40 are also expressed from the internal promoters, which are named according to their length in kilodaltons (Muntoni et al., 1995; Daoud et al., 2009; Naidoo and Anthony, 2020). Full-length dystrophin is expressed in a tissue-specific manner through the proximal promoters in the brain, whereas short dystrophin isoforms are expressed through distant upstream promoters (Boyce et al., 1991). In mouse, dystrophin was reported to be localized in the neuronal postsynaptic specializations of cerebellar Purkinje’s cells and in cerebral cortical pyramidal cells (Lidov et al., 1990). Moreover, human DMD brain study also reported deficiency of dystrophin in the postsynaptic densities (PSD) of the brain (Kim et al., 1995). Dp140, Dp71, and, an alternatively spliced shortest isoform, Dp40 are reported to be expressed in the various brain regions including the cerebral cortex, cerebellum, and hippocampal dentate gyrus (Greenberg et al., 1996; Lidov and Kunkel, 1997; Daoud et al., 2009; Tozawa et al., 2012; Naidoo and Anthony, 2020). The expression of dystrophin and short isoforms in the crucial brain regions indicates their crucial role for higher-order cognitive functioning (Doorenweerd, 2020). Dystrophin’s localization and interaction in the brain regions for performing higher-order functions necessitate the domain-wise investigation according to the population dynamics (Wicksell et al., 2004; Doorenweerd, 2020; Tyagi et al., 2020).

Similar genotype–phenotype correlation studies have previously associated the neurological and intellectual outcomes in various neurological disorders in which genetic screenings indicated specific pathologies (Anand et al., 2007, 2012a, 2012b, 2015; Sharma et al., 2012; Goyal et al., 2014). However, in Indian children with DMD, studies elucidating the cognitive and neuropsychological functioning are scarce. A South Indian study reported significantly lower IQ in DMD subjects compared to the normative data (Perumal et al., 2015). However, the small sample size, absence of a matched control group, and less coverage of neuropsychological domains were major limitations of this study. Hence, we studied the cognitive function of DMD children by assessing various cognitive and neuropsychological domains and compared these results with a matched control group.

## Materials and Methods

### Participants

The current study included 69 children with DMD. Sixty-six age-, sex-, and education-matched children and adolescents in the age group of 6–16 years served as a control group. The inclusion criteria included a diagnosis of DMD based on clinical features and genetic tests. The exclusion criteria included children having psychiatric co-morbidities including autism spectrum disorders, attention deficit hyperactivity disorder (ADHD), and epilepsy, etc. The study was approved by the Institutional Ethical Committee (IEC). Written informed consent was obtained from the parents or legal guardians (as children were minor) before inclusion in the study. The study was conducted in the Neuroscience Research Lab of Postgraduate Institute of Medical Education and Research, Chandigarh, India. The pathogenic variants in the *DMD* gene were obtained by multiplex ligation-dependent probe amplification as described earlier in our cohort (Tyagi et al., 2019a).

### Assessment of Cognitive Functions

#### Intelligence

Malin’s Intelligence Scale for Indian Children (MISIC) [an Indian adaptation of Wechsler Intelligence Scale for Children (WISC)] was used to assess intelligence (Malin, 1970). It measures both verbal and performance IQ. *Verbal subsets* included tests of information, comprehension, arithmetic, analogies, and similarities; vocabulary; and Digit Span (DS) test, while *performance subsets* included the picture completion, block designing, coding, maze, and object assembly. Raw scores for each case were converted into age-adjusted test quotients using the normative data. Verbal IQ (VIQ) and performance IQ (PIQ) were obtained by averaging the tests of verbal subsets and performance subsets, respectively. The average score of VIQ and PIQ was used to obtain the global intelligence quotient (IQ).

### Assessment of Specific Cognitive Domains

Subjects with IQ > 69 were carried forward for a detailed assessment of specific cognitive domains. Details of test batteries and corresponding cognitive and neural correlates are provided in Supplementary Table 1.

#### Rey Auditory Verbal Learning Test

Rey Auditory Verbal Learning Test (RAVLT) was used to measure learning, working, short- and long-term memory, susceptibility to interference, serial positioning effect, recognition memory, and verbal memory efficiency index. In the present study, the adapted version for the Indian population was used (Kar et al., 2004). A list of 15 nouns (list A) was read aloud during five consecutive trials. In each trial, an interval of 1 s was maintained between presentations of two nouns. A subsequent list (list B) of 15 words were presented after list A (trials 1–5) as an interference. The subject was asked to recall the list A immediately after the list B task. After 20 min, the subject was then instructed to recall list A again to access the long-term verbal memory. To assess the recognition, 30 words (15—list A and 15 other words) were presented and the subject was asked to recognize list A out of 30 words. Omissions (from list A) and commissions (non-list A words) were recorded. The number of words correctly recalled in each RAVLT trial as well as in immediate (after list B) and delayed recall (after 20 min) tasks were used as scores. Learning capacity was assessed by summing the total list A words recalled over five trials. The subject’s susceptibility to interference was obtained through proactive interference (PI) and retroactive interference (RI). PI reveals the negative effect of previously learned material in the acquisition or recall of new information (Vakil et al., 2010). Similarly, RI reveals the negative effect of new learning in recalling previously learned information. Serial positioning effect was assessed by obtaining primacy (first one-third of 15 words), middle (middle one-third of 15 words), and recency (last one-third of 15 words) scores of list A trials 1–5 (Boone et al., 2005). RAVLT Memory Efficiency Index (MEI) was obtained by using all RAVLT components based on a previous study (Ricci et al., 2012). Calculations to obtain various factor indexes from RAVLT data have been depicted in Supplementary Table 2.

#### Rey–Osterrieth Complex Figure Test

It was administered to measure visual learning, short- and long-term visual memory, and visuo-constructive ability of the child. This figure consists of a complex design with multiple subcomponents, which was placed in front of the subject. For visuo-constructive ability, the subject was instructed to draw the figure on the paper with freehand. For visual memory, the complex figure was recalled twice, an immediate recall (IR) immediately after copying task and delayed recall (DR) after 30 min. Scoring was done based on the accuracy and placement of the components of a complex figure based on a previous study (Duley et al., 1993).

#### Stroop Color and Word Test

Stroop test, a measure of executive functioning, was used to record response inhibition, selective attention, and cognitive flexibility (Golden, 1975). The Stroop Color and Word Test (SCWT) consists of a 5-by-20 matrix of words representing three colors (red, blue, and green) each in three sheets to record neutral, congruent, and incongruent tasks. The first sheet consisted of 100 (5 × 20) names of three colors (red, blue, and green) printed only in black color to record the neutral task. The second sheet consisted of 100 (5 × 20) XXXX symbols printed in three colors (red, blue, and green) to record congruency. The third sheet consisted of 100 (5 × 20) color names, which were printed in another color (red/blue/green), e.g., red-written word printed in blue/green color as an incongruent task. Subjects were instructed to name the color instead of reading the written words down the column. Forty-five seconds were provided to finish each task. The number of words read in each sheet was considered the score of a participant. The last task has an interference component because it requires the participant to override or inhibit a reading response. This test measures the ease with which a person can shift his or her perceptual set to conform to changing demands and inhibit the usual response from interfering with the unusual one. The interference component (also called Stroop effect) was calculated based on the following formula: Stroop effect = SCWT color (raw) − SCWT color − word (raw) (Jensen and Rohwer, 1966).

#### Children’s Color Trail Test A & B

Children’s Color Trail Test (CCTT) is a measure of sustained attention and is found to be very sensitive to brain damage (Llorente et al., 2003). It has two parts, part A and part B. In trail A, circles were numbered 1 to 15 in two colors: yellow and pink. The subject was required to connect the numbers 1 to 15. Trail B consisted of the 1 to 15 numbered circles in two colors: pink and yellow, and the subject was required to link the numbered circles with alternative colors. The time taken to complete the task and interference index was considered an outcome measure. The interference component was calculated based on the following calculation: CCTT interference index = (CCTT2 time raw score − CCTT1 time raw score)/CCTT1 time raw score.

#### Color Cancellation Test

This test is a measure of focused attention, accurate visual scanning, and activation or inhibition of rapid response (Llorente et al., 2003). A sheet consisting of 150 circles in five different colors (red, yellow, blue, black, and gray) was presented. The subject was required to cancel only the red and yellow circles as fast as possible. The time taken to complete the task was used as scores.

#### Controlled Oral Word Association Tests

It measures phonemic verbal fluency. The original Controlled Oral Word Association Tests (COWA) used alphabets starting with FAS to generate words (Ruff et al., 1996). It has been adapted for the Indian population (Kar et al., 2004). Subjects were asked to generate words beginning with Ka, Pa, and Ma as many as possible in 1 min. Scoring was done according to acceptable new word formation over three trials.

#### Animal Naming Test

The animal naming test is a measure of the category verbal fluency. The animal naming test requires the subject to generate the names of animals as many as possible in 1 min. Scoring was done according to the generation of newly formed words (Regard et al., 1982).

#### Visual Recognition Test

This test has been selected from the NIMHANS battery for children manual (Kar et al., 2004). It is a measure of visual agnosia and the capacity of the subject to recognize subjects visually. The test consists of a card with 10 pictured objects that were required to be recognized and named by the subject.

### Statistical Analysis

SPSS (ver. 21.0) was used to perform statistical analysis. Kolmogorov–Smirnov (KS) test was performed to check the normality of data variables. A comparison between two independent normally distributed data was carried out by independent Student’s *t*-test with equal or unequal variance (Welch’s correction). For non-normally distributed data, a non-parametric Mann–Whitney *U* test was performed. A *p*-value ≤ 0.05 was considered to be significant for testing the hypothesis. The study seeks to show that working memory underlies cognitive deficits in this population. However, this cannot be demonstrated by simply showing that working memory tasks are correlated with all the other tasks. Hence, regression analyses and domain factor scores were derived to demonstrate dissociation by showing that other domains do not perform as consistently as working memory in predicting other skills. Linear regression was performed and scales of working memory functioning were considered dependent variables (RAVLT1, Digit Span Backward, RAVLT MEI, WMI, and Stroop task color word scale). The forward selection method was chosen for analysis. Principal component analysis was performed followed by varimax rotation with Kaiser normalization. Eigenvalue > 1 was considered. The factor structure of DMD cases and controls was analyzed. An absolute value below 0.3 was considered a coefficient display.

## Results

### Demographic Variables

Demographic details of DMD and control subjects have been provided in Table 1.

**TABLE 1:** Demographic details of study participants.

Demographic parameter	DMD (*n* = 69)	Control (*n* = 66)	*p*-value
Age, mean (SD)	10.78 (2.65)	10.37 (2.1)	0.371
Education level, mean (SD)	4.22 (2.3)	4.21 (2.05)	0.971
Income per month (INR)	34,755 (48621)	40,131 (52555)	0.626
Age of onset (years), mean (SD)	4.16 (2.10)	NA	NA
Disease duration	6.36 (2.7)	NA	NA

### Intellectual Functioning in DMD

General intellectual abilities of DMD subjects were categorized based on ICD-10 guidelines. Out of 69 DMD patients, 48 (69.56%) had adequate intelligence (IQ > 84) and 16 (23.18%) demonstrated borderline intelligence (IQ 70–84). Only 5 (7.24%) subjects demonstrated intellectual disability (IQ < 70), among which 1(1.44%) had moderate and 4 (5.79%) had mild intellectual disability. Similarly, verbal IQ was found to be impaired in 8.69% DMD cases whereas 5.79% demonstrated impairment in performance IQ. In the control group, all had adequate intelligence.

Duchenne muscular dystrophy subjects revealed a mean IQ of 90 (SD = 14.54; range = 42–123), VIQ of 87 (SD = 14.71), and PIQ of 93 (SD = 16.29) shown in Table 2. Verbal discrepancy (VIQ-PIQ) of −6.50 (SD = 10.80) was observed compared to −2.07 (SD = 15.51) of control with significant difference (*t* = −1.918, *p* = 0.05). Seventy-eight percent of DMD participants exhibited less VIQ than the PIQ.

**TABLE 2:** Comparison of general intelligence among DMD and control population.

General intelligence	DMD Mean (SD)	Control Mean (SD)	*t*-value	*p*-value
Verbal intelligence quotient	87 (14.71)	108 (14.30)	−8.559	<0.001
Performance intelligence quotient	93 (16.29)	110 (13.00)	−6.695	<0.001
Intelligence quotient	90 (14.54)	109 (11.33)	−8.571	<0.001
VIQ-PIQ	−6.50 (10.80)	−2.07 (15.51)	−1.918	0.058

### Assessment of Specific Cognitive Abilities

Specific cognitive abilities were assessed only in DMD cases (*n* = 64) with IQ ≥ 70 and the performance was compared with healthy controls. Both groups were matched on age and education.

#### Intelligence

The mean IQ of DMD and control groups were 92 (SD = 11.48) and 109 (SD = 11.33), respectively, and the differences were significant concerning VIQ and PIQ. Factor index analysis in the DMD subjects revealed poor performance in the Verbal Comprehension Index (*p* < 0.001), Working Memory Index (*p* < 0.001), and Perceptual Reasoning Index (*p* < 0.001) in comparison to the control. Scores have been depicted in Table 3.

**TABLE 3:** Comparison of subsets of general intelligence between DMD and control groups.

MISIC subtests	DMD Mean (SD)	Control Mean (SD)	*t*-value	*p*-value
**Verbal subtests**				
Information	12.08 (4.57)	15.58 (4.96)	–4.179	<0.001
Comprehension	9.86 (4.73)	15.20 (5.76)	–5.782	<0.001
Arithmetic	7.80 (2.75)	10.58 (2.55)	–5.959	<0.001
Digit span	7.83 (2.46)	9.03 (2.37)	–2.830	0.005
Similarity	10.27 (4.96)	15.11 (5.12)	–5.163	<0.001
**Performance subtests**				
Picture completion	6.94 (2.73)	9.75 (2.66)	–5.895	<0.001
Block design	16.02 (12.84)	21.69 (11.75)	–2.522	0.013
Coding	27.91 (15.82)	38.85 (13.03)	–4.109	<0.001
Maze	14.98 (10.12)	17.68 (1.88)	–2.013	0.048
Object assembly	7.750 (7.19)	11.914 (5.52)	–2.692	0.009
**Factor indexes**				
VCI	255 (57.14)	338 (50.78)	–8.685	<0.001
WMI	172(27.41)	201 (29.37)	–5.907	<0.001
PRI	165 (54.16)	205 (47.26)	–4.498	<0.001
FOD	172 (27.41)	201 (29.80)	–5.795	<0.001

#### Sustained and Focused Attention

We used CCTT and CCT to measure sustained and focused attention. The mean CCTT completion time score between DMD and control groups was obtained. DMD group took more time (*p* < 0.001) in completing CCTT-A, a measure of psychomotor sequencing, visual tracking, processing speed, and graphomotor skills, indicating poor performance in the attention domain. Similarly, CCTT-B, representing divided attention, set-switching, inhibition, and working memory/sequencing, was also poorly performed by the DMD group (*p* < 0.001). However, the CCTT interference score was found to have significant (*p* = 0.05) differences in comparison to the control group. CCT, a measure of selective attention and visual scanning, was poorly performed by DMD subjects (*p* < 0.001). Errors made in the CCT task were comparable to the control group.

#### Executive Functioning

Executive functioning was measured by COWA, Animal Naming Test (ANT), and SCWT task performance. COWA performance indicates the subject’s phonemic knowledge and language fluency. The mean retrieval in COWA was 4.29 (SD = 3.00) in comparison to the control group 6.16 (SD = 2.75) with significant differences (*t* = −3.675, *p* < 0.001). In the semantic fluency assessment, DMDs demonstrated a raw score of 8.95 (SD = 3.49) whereas the raw score of controls was 11.48 (SD = 4.08) with significant differences (*t* = −3.804, *p* < 0.001) as depicted in Table 4.

**TABLE 4:** Comparison of neuropsychological variables between DMD and control groups.

	Control		DMD		*t*-value/*z*-value	*p*-value	
NP	Mean	SD	Mean	SD			Cohen’s *D*
COWA-total	18.52	8.27	12.88	9.01	−3.690	<0.001***	0.65
COWA avg	6.16	2.75	4.29	3.00	−3.675	<0.001***	0.65
ANT	11.48	4.02	8.95	3.49	−3.804	<0.001***	0.67
							
RCFT-copy	33.01	3.78	28.55	8.77	−3.174	0.002**	0.66
RCFT-IR	25.38	6.86	19.50	9.17	−3.606	0.001**	0.73
RCFT-DR	24.71	6.37	19.21	9.01	−3.460	0.001**	0.70
							
Stroop-W	58.12	12.75	48.02	17.54	−3.361	0.001**	0.66
Stroop-C	45.83	9.84	35.16	13.46	−4.617	<0.001***	0.91
Stroop-CW	28.03	8.53	20.90	7.75	−4.551	<0.001***	0.87
Stroop effect	17.50	7.58	13.98	9.44	−2.126	0.036*	0.41
							
CCTT1	34.13	16.05	62.00	42.61	4.212	<0.001***	–0.87
CCTT2	67.28	24.85	100.78	50.41	4.138	<0.001***	–0.84
CCTT interference	1.15	0.78	0.80	0.56	−1.951*	0.051	0.52
CCT	86.75	33.28	127.72	53.75	4.584	<0.001***	–0.92
VRT	9.44	0.73	8.22	1.21	−6.474	<0.001***	1.22

Number of control (*n* = 64) and DMD subjects assessed in various scales: COWA and ANT (*n* = 64), RCFT (*n* = 44), Stroop test (*n* = 49), CCTT and CCT (*n* = 46), and VRT (*n* = 55). Level of significance *P < 0.05; **P < 0.01; ***P < 0.001.

#### Performance on SCWT

Scores of SCWT revealed a poor DMD performance on all measures of SCWT as represented in Table 4. Neutral word task score indicating poor personal tempo and speed showed significant poor performance among DMD subjects (*t* = −3.361; *p* = 0.001). The congruent task, a measure of color perception, has also been found to be affected. The incongruent task, a measure of response inhibition and negative priming, was also affected. Stroop effect was calculated and DMD subjects were found to be susceptible to interferences (*p* = 0.036) in comparison to the control group. It indicated poor cognitive control and selective attention of participants.

We used the Visual Recognition Task as a measure of parietal lobe functioning. DMD subjects recognized a reduced number of pictured objects in comparison to the control group (Table 4). Block designing (BD) task was used to measure visuo-constructive abilities in DMD. Though the test quotient revealed poor visual-motor coordination (*t* = −2.522, *p* = 0.013), we formed an efficiency index by using test parameters to obtain a motor component-free analysis. There was no difference between DMD and control cases on BD efficiency (0.183). The time taken to complete the BD was comparable to the control subjects (*p* = 0.551). However, the level of complexity of block designing tasks that also require working memory manipulation of resources was found to be affected (*p* = 0.011). Maze scores were analyzed to measure visuospatial ability, a crucial element of working memory. DMD subjects were found to be poor in comparison to the control group. The test quotient (*p* < 0.001) and the time taken in completing the maze task was also significantly different (*p* = 0.024). Similarly, errors made in the maze performance was also significantly higher in the DMD group (*p* = 0.009).

We used RAVLT, DST, and Rey–Osterrieth complex figure test (RCFT) to measure verbal and visual memory changes in DMD and control groups. Comparison of RAVLT learning trials between DMD and control groups showed significant differences in trial 1, *U* = 1,570, *p* = 0.015 with a mean (SD) value of 6.24 (2.33) for the DMD group compared to 7.15 (1.92) for the control group, checking these units properly. In trial 1, 27% of DMD subjects showed impairment. The learning trend of DMD subjects was similar to the control group as evidenced by similar performance in each learning trial after trial 1.

**FIGURE 1:**
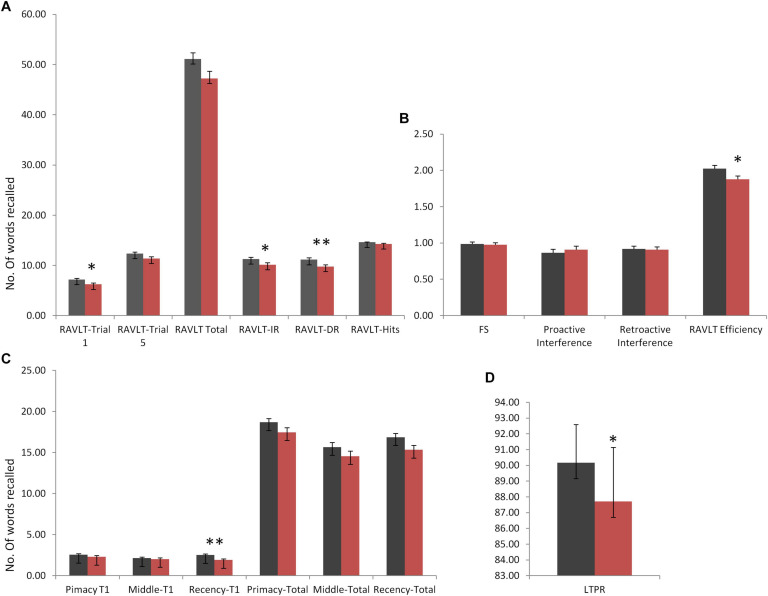
Rey Auditory Verbal Learning Test (RAVLT) data represents trends of verbal memory impairment in Duchenne muscular dystrophy (DMD) subjects. **(A)** A significant difference in trial 1, immediate and delayed verbal memory. **(B)** No difference was observed in the susceptibility to interferences through proactive interference (PI) and retroactive interference (RI) scores. RAVLT-Memory Efficiency Index (MEI) was found to have cut-off values close to the patients of behavioral variant frontotemporal dementia (bvFTD). **(C)** Among serial positioning factors, recency T1 score was found to be reduced significantly. **(D)** Long-term percent retention of DMD subjects was also affected. All results expressed as mean ± standard error of mean (SEM). Data was statistically analyzed using SPSS 16.0 by independent *t*-test/non-parametric test (Mann–Whitney test) as applicable. *p < 0.05, **p < 0.01, ***p < 0.001.

Rey Auditory Verbal Learning Test immediate and delayed recall scores were observed to be significantly different in DMD subjects (IR: *U* = 1,651, *p* = 0.042; DR: *U* = 1,525, *p* = 0.009), indicating poor short- and long-term verbal memory. Moreover, DMD subjects also demonstrated a significant reduction in the long-term percent retention (LTPR) (*U* = 1,537; *p* = 0.010) as depicted in Figure 1.

No differences in the learning capacity were observed in the DMD subjects (*p* = 0.071). An analysis of serial positioning effect revealed poor recency in DMD cases. This measure of immediate memory significantly differed in comparison to the control group (*U* = 1,478, *p* = 0.004). However, primacy and middle words recalling were intact and comparable to the control group. Factor scores including proactive interference, retroactive interference, and forgetting speed from the RAVLT data are compiled in Table 5, which showed no difference when compared to control subjects. RAVLT Memory Efficiency Index (RAVLT-MEI) was calculated by incorporating various RAVLT components as shown in Supplementary Table 2. An RAVLT memory efficiency index of 1.8 was significantly lower among DMD subjects than the control group with RAVLT-MEI of 2.0 (*U* = 1,516, *P* = 0.008). Impairment in the RAVLT trials has been depicted in Table 5.

**TABLE 5:** Comparison of RAVLT variables between DMD (*n* = 63) and control group (*n* = 66).

Cognitive domain and neuro psychological battery	Neuro psychological battery variables	DMD Mean ± SD	DMD Mean Rank	Control Mean ± SD	Control Mean rank	*Z*-value	Mann– Whitney *U*	*p*-value
RAVLT	RAVLT-trial 1	6.24 (2.33)	56.91	7.15 (1.92)	72.72	–2.424	1,570	0.015
•Verbal learning	RAVLT-trial 5	11.38 (2.89)	58.89	12.33 (2.36)	70.83	–1.832	1,694	0.067
•Short-term verbal memory	RAVLT-learning capacity	47.24 (11.52)	58.92	50.97 (9.90)	70.80	–1.805	1,696	0.071
•Long-term verbal memory	RAVLT-IR	10.16 (3.23)	58.20	11.29 (2.93)	71.49	–2.031	1,651	0.042
	RAVLT-DR	9.78 (3.04)	56.20	11.14 (3.11)	73.40	–2.626	1,525	0.009
	RAVLT-hits	14.27 (1.18)	58.88	14.61 (0.76)	67.70	–1.648	1,703	0.099
	Omission	0.75 (1.19)	68.33	0.38 (0.72)	59.11	–1.722	1,690	0.085
	Commission	0.87 (2.10)	66.80	0.52 (1.07)	60.50	–1.147	1,782	0.251
	LTPR	87.71 (27.25)	56.40	90.23 (20.01)	73.21	–2.561	1,537	0.010
RAVLT	Primacy-T1	2.30 (1.29)	61.56	2.52 (1.08)	68.29	–1.061	1,862	0.289
•Serial positioning effect	Middle-T1	2.03 (1.22)	63.06	2.12 (1.13)	66.85	–0.593	1,957	0.553
	Recency-T1	1.92 (1.15)	55.46	2.53 (1.15)	74.11	–2.916	1,478	0.004
	Primacy-total	17.46 (4.50)	60.92	18.61 (3.71)	68.89	–1.215	1,822	0.224
	Middle-total	14.56 (5.04)	61.51	15.59 (4.68)	68.33	–1.039	1,859	0.299
	Recency-total	15.33 (4.35)	58.94	16.85 (3.87)	70.79	–1.806	1,697	0.071
RAVLT	Proactive interference	0.91 (0.41)	65.68	0.86 (0.28)	64.35	–0.203	2,036	0.839
•Susceptibility to interferences	Retroactive interference	0.91 (0.31)	61.66	0.92 (0.17)	68.19	–1.008	1,869	0.313
	Forgetting speed	0.98 (0.22)	60.57	0.99 (0.18)	68.19	–1.171	1,803	0.242
	RAVLT efficiency	1.8 (0.36)	56.06	2.0 (0.27)	73.54	–2.655	1,516	0.008
								

#### DST

The Digit Span subtest of MISIC provides an opportunity to understand the attentional loop of working memory. The DMD group’s Digit Span forward performance was statistically comparable to the control group (*p* = 0.159), indicating a normal auditory short-term memory and simple verbal expression. However, Digit Span Backward task performance revealed an inefficient working memory domain in comparison to the control group (*p* = 0.013). Differences between the DSF and DSB tasks have been suggested to tease out attention from the working memory (Hale et al., 2002). The fraction of attention was obtained by the pooled Digit Span score and it was found to be comparable to the control group (*U* = 1,663, *p* = 0.067). It also indicated the crucial role of working memory in the DMD cognitive functioning.

#### Performance on RCFT

Compared to control group, DMD patients demonstrated a significantly lowered score on the RCFT copy (*t* = −3.174, *p* = 0.002), immediate recall (*t* = −3.606, *p* = 0.001), and delayed recall (*t* = −3.460, *p* = 0.001) as depicted in Table 4.

#### Estimating the Effect Size

Cohen’s *D* values were calculated for each parameter. Among the RAVLT parameters, recency T1 (effect size, ES = 0.53) and RAVLT efficiency index (ES = 0.63) showed a medium effect size. RCFT data showed medium effect in the RCFT copy (ES = 0.66), RCFT-IR (ES = 0.73), and RCFT-DR (ES = 0.70). Executive functioning domains including Stroop congruent (ES = 0.91) and incongruent task (ES = 0.87), CCTT-A (ES = −0.87), CCTT-B (ES = −0.84), and CCT (ES = −0.92) tasks revealed large ES. The effect size for Visual Recognition Test (VRT) was found to be very large (ES = 1.22).

### Association of Working Memory Components With Various Cognitive Domains

**FIGURE 2:**
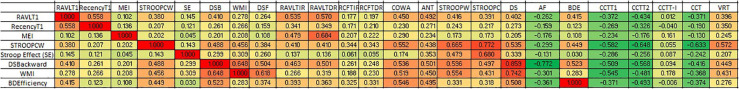
Correlation heat map showing association of working memory with other cognitive domains.

Correlation between various cognitive domains showed that working memory components have been highly correlated with the executive functioning domains. It also revealed the flow of domain dysfunctions primarily due to working memory alterations. The short-term verbal memory revealed a significant positive correlation with working memory components especially RAVLT-T1 (Spearman rho = 0.535; *p* < 0.001). However, verbal long-term memory correlated strongly with the RAVLT memory efficiency index (Spearman rho = 0.684; *p* < 0.001). The data indicate that learning in RAVLT requires efficient working memory. However, long-term memory formation is an effect of various components including working memory, retention, and recognition. However, in comparison to verbal memory, visual memory correlated less with the verbal working memory. Moreover, the more complex cognitive domains such as executive functioning have been strongly associated with the working memory components as depicted in Figure 2.

Linear regression analysis was performed considering RAVLT1 and Digit Span Backward task as dependent variables. The model with RAVLT delayed recall, and Stroop neutral task revealed a *B* value of 1.103. A higher *B* value of 2.104 was observed in a model with RAVLT delayed recall alone. All of the predictors in both models revealed statistical significance. Adjusted *R* square was 0.335 for the model as presented in Table 6.

**TABLE 6:** Linear regression of DMD neuropsychology data.

Dependent variable	Model	Unstandardized coefficients		Standardized coefficients	*t*-score	*P*-value
		*B*	Std. error	Beta		
RAVLT1*	(Constant)	2.104	0.810		2.597	0.011*
	RAVLTDR	0.437	0.071	0.545	6.166	<0.001***
	(Constant)	1.103	0.867		1.272	0.207
	RAVLTDR	0.356	0.075	0.444	4.760	<0.001***
	STROOPW	0.035	0.013	0.251	2.693	0.008**
DSTB	(Constant)	0.578	0.536		1.079	0.290
	STROOPW	0.058	0.010	0.762	6.112	<0.001***
	(Constant)	2.877	0.922		3.120	0.004**
	STROOPW	0.040	0.010	0.527	3.850	0.001**
	CCTT2	–0.016	0.006	–0.398	–2.907	0.007**
RAVLT1	(Constant)	4.755	0.740		6.424	<0.001***
	COWAAVG	0.379	0.108	0.561	3.519	0.002**
	(Constant)	11.628	3.112		3.736	0.001**
	COWAAVG	0.465	0.107	0.687	4.334	<0.001
	MISICVIQ	–0.079	0.035	–0.359	–2.264	0.032*
	(Constant)	10.986	2.907		3.778	0.001**
	COWAAVG	0.408	0.103	0.604	3.970	0.001**
	MISICVIQ	–0.104	0.034	–0.473	–3.035	0.006**
	RAVLTIR	0.284	0.126	0.351	2.255	0.033*

Level of Significance *P < 0.05; **P < 0.01; ***P < 0.001.

#### Principal Component Analysis

**FIGURE 3:**
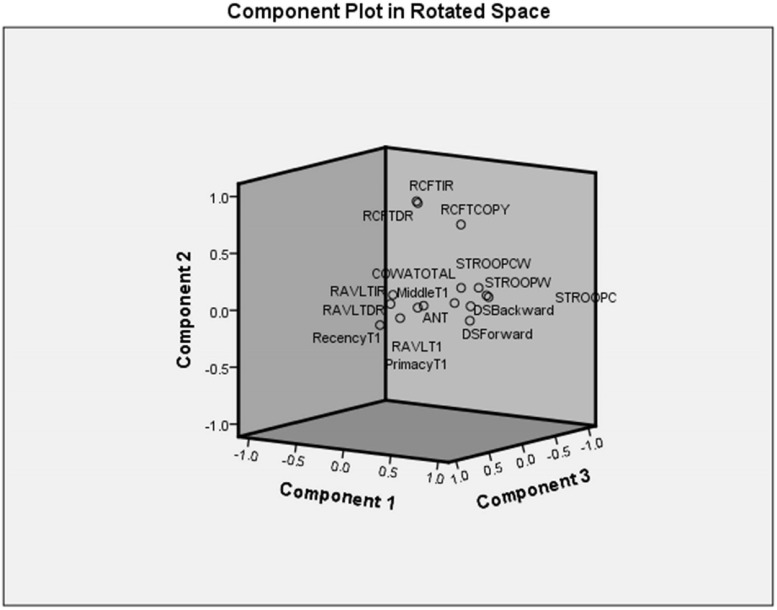
Component plot in rotated space.

Principal component analysis extracted five components (eigenvalues > 1) that explained 75% variance. Table 7 shows the factor structure in five components with higher eigenvalues. The component plot is provided in Figure 3.

**TABLE 7:** Rotated component matrix^*a*^.

	Component				
	1	2	3	4	5
STROOPC	0.813				
STROOPW	0.801				
STROOPCW	0.749				
DSBackward	0.730				
DSForward	0.711				
COWATOTAL	0.595				
ANT	0.560				
RCFTIR		0.934			
RCFTDR		0.928			
RCFTCOPY		0.738			
RecencyT1			0.805		
RAVLTIR			0.770		
RAVLTDR			0.761		
MiddleT1				0.926	
RAVLT1				0.637	
PrimacyT1					0.923

*Extraction method: principal component analysis. Rotation method: Varimax with Kaiser Normalization^*a*^. ^*a*^Rotation converged in five iterations.*

## Discussion

Inadequate intelligence in approximately 30% DMDs (Cotton et al., 2001) and poor functioning of various neuropsychological domains necessitate immediate rehabilitation strategies. In contrast to previous reports, approximately 7% of our DMD cohort revealed intellectual disability (IQ < 70). DMD subjects had a mean IQ of 90 (14.54) indicating average intelligence. There are inconsistent findings in previous studies that reported different intelligence quotients in different ethnic populations. A recent study conducted in South Indian DMD patients reported low average intelligence (mean IQ = 88.5 ± 13.18), which also indicates population-based investigation and intervention regime (Perumal et al., 2015). Verbal components remained vulnerable in ours as well as the South Indian cohort as VIQ was found to be affected more than the performance IQ score.

Assessment of verbal memory through RAVLT revealed deficits in immediate and delayed verbal memory in our cases. The poor performance on RAVLT trial 1 revealed deficits in the short span memory process, which may lead to difficulties in the maintenance and manipulations of verbal information. However, we found an adequate trend of learning after trial 1 till trial 5 indicating a normal learning trend in the DMD subjects. Thus, multiple exposures (rehearsal) of information may help DMD subjects to perform complex cognitive performance. RAVLT memory efficiency index (RAVLT-MEI) (Ricci et al., 2012), which combined measures of encoding and retention, revealed a marked reduction of scores. Ricci et al. (2012) reported the cut-off range of 1.2 and 1.9 to differentiate the patients with Alzheimer’s disease/behavioral variant frontotemporal dementia (bvFTD) and bvFTD/normal controls to evaluate the prognostic impact of this factor index. Similarly, in our study, RAVLT-MEI value for DMD was found to be 1.8, suggesting lower levels of verbal retention and encoding, similar to the patients with frontotemporal dementia, which emphasizes the role of frontal and temporal lobes in DMD subjects (Figure 1). RAVLT-MEI score may also indicate the working memory resources of the DMD brain. The DMD group showed a normal trend in the acquisition of the list of words learned initially (primacy) and middle in order. However, recently, learned information was inadequate, indicating poor working memory as also evidenced by RAVLT trial 1 performance and MEI (Figure 1). The arithmetic subtest and Digit Span Backward task further indicated deficits in the attention loop and working memory axis.

Along with the poor performance of verbal working memory, DMD subjects also demonstrated poor visual memory, visuo-constructive, and visuospatial abilities. However, the role of motor restrictions in these tasks necessitated the investigation of motor-free efficiency indexes negating the muscular effects. Therefore, block designing efficiency scores were introduced in this study, which demonstrated a normal visuo-constructive ability in DMD. Also, DMD subjects were able to normally switch between the neural circuits in the presence of the appropriate stimulus. Previous studies have observed a less consistent poor visual memory (Billard et al., 1992). In contrast, our study subjects demonstrated poor visual immediate and long-term memory. A likely explanation could be the variable mutation location and affected dystrophin isoforms in our cohort (Tyagi et al., 2019a). Furthermore, linear regression analysis of working memory alterations through RAVLT1 was associated with the long-term verbal memory. It suggested that working memory loss also affects verbal long-term memory. We previously associated and validated the working memory alterations with Dp140 isoform through computational cognitive modeling (Tyagi et al., 2020). Recent radiological investigation reported extensive abnormalities in white matter of DMD cases affecting Dp140 isoform (Preethish-Kumar et al., 2020). White matter microstructure has previously been associated with working memory (Tokariev et al., 2019). The contribution of altered working memory in affecting other neuropsychological domains was underexplored in DMD.

An assessment of visual working memory could have shed more light on the poor visual immediate and long-term memory in DMD. Previously, Cowan et al. (2010) demonstrated that visual working memory capacity highly correlates with intelligence in children. Underperformance in the CCTT task indicated difficulty in the alternating and sustained visual attention, perceptual tracking, simple sequencing, and psychomotor speed. The DMD group took a longer time in finishing CCTT-A and CCTT-B. Semantic (ANT) and phonemic (COWA) verbal fluency scores were not similar to the scores obtained in the control group, indicating poor verbal ability and executive control. Poor Stroop Color and Word Task (SCWT)-Word (W) performance of the DMD group corresponds to personal tempo, speech motor problems, and learning disabilities. SCWT color and color word performance was found to be altered in the DMD group, which reflected poor health of the primary visual cortex since it is responsible for the spatial selectiveness and color perception (Johnson et al., 2001; Solomon and Lennie, 2007). Factor indexes such as RAVLT-memory efficiency index, primacy and recency factors, proactive and retroactive interferences, LTPR, block designing efficiency, and attention fractions provide evidence of alterations as well as strengths of DMD brain processes especially in disorders demonstrating motor restrictions.

Working memory is strongly associated to the attentional control as a part of central executive. Luria (1973) provided three model components of a working brain in his book, which consisted of memory system, attention system, and activation system. Processing of attention requires effective processing of four sub-components, which include working memory as a central component along with competitive selection, top-down sensitivity control, and automatic bottom-up filtering for salient stimuli (Knudsen, 2007). Information obtained from the environment is filtered before its access to the working memory based on appropriate stimulus. This phenomenon is referred as bottom-up filtering for salient stimuli. However, based on the strength and quality of the signals, information is selected for getting access of working memory. This phenomenon is referred as competitive selection. These two processes are crucial before processing of appropriate information before further encoding. Limited working memory capacity plays an essential role of presenting and manipulating the information in conjunction to the activation of brain areas to support the attentional control (Knudsen, 2007). Thus, working memory may contribute to the formation of short-term memory as well as it is inextricably inter-related to attentional processing. Our study results propose a deeper investigation to study the molecular aspects of understanding dystrophin isoform-associated mechanism of working memory regulations. Moreover, interventions at the level of working memory axis (working memory, attention, and visuospatial functioning) may probably improve the neuropsychological impairments in DMD.

## Conclusion

Duchenne muscular dystrophy subjects showed poor general intellectual abilities as compared to the normal population. Among the cognitive domains analyzed, working memory functioning seems to affect DMD neuropsychological profile. It can be used as a potential target for rehabilitation strategies. Novel factor indexes including RAVLT-MEI demonstrated poor verbal memory efficiency. The DMD group also underperformed on attention, executive functioning, visual memory, verbal fluency, and sustained and focused attention tasks.

## Data Availability Statement

The raw data supporting the conclusions of this article will be made available by the authors, without undue reservation.

## Ethics Statement

The studies involving human participants were reviewed and approved by Institutional Ethics Committee, Postgraduate Institute of Medical Education and Research, Chandigarh, India. Written informed consent to participate in this study was provided by the participants’ legal guardian/next of kin.

## Author Contributions

AA was responsible for the conceptualization and editing and serves as the grant PI. RT was responsible for co-conceptualization under supervision, genetic and neuropsychological data acquisition, experiments and analysis, statistical analysis, drafting, and editing the manuscript. HA was responsible for neuropsychological data acquisition. MG is the co-supervisor of the first author. MM was responsible for supervision of neuropsychological assessment, analysis, and validation of data and final approval of the manuscript. All authors contributed to the article and approved the submitted version.

## Conflict of Interest

The authors declare that the research was conducted in the absence of any commercial or financial relationships that could be construed as a potential conflict of interest.
