# Supplementary material for: Working Memory Alterations Plays an Essential Role in Developing Global Neuropsychological Impairment in Duchenne Muscular Dystrophy
Source: Front Psychol. 2021 Jan 15;11:613242. doi: 10.3389/fpsyg.2020.613242 (PMC7843380; doi:10.3389/fpsyg.2020.613242)
Supplement: Supplementary file 1 [file Table_1.DOC]

Supplementary Table 1: Details of neuropsychological tests used to assess the functioning of specific cognitive domains and their neuroanatomical correlates.

| **Cognitive Domain** | **Sub- Domain** | **Test** | **Neural correlates** |
| --- | --- | --- | --- |
| **Memory** | Verbal-Memory (Short & long term) | Rey Auditory Verbal Learning Test (RAVLT) | ***Temporal Lobe*** |
|  | Visual-Memory (Short & long term) | Rey-Osterrieth Complex Figure Test (RCFT) | ***Temporal Lobe*** |
|  | Working Memory | RAVLT T1, DSB, WMI, Recency T1 | **Pre Frontal Cortex** |
| **Attention** | Focused Attention | Children’s Color Trail Test | ***Frontal Lobe*** |
|  | Sustained Attention | Color Cancellation Test |  |
|  | Attention Fraction | DSF-DSB/DS |  |
| **Executive Functioning** | Response Inhibition/ Cognitive Flexibility | Stroop Color And Word Test | ***Anterior Cingulate Cortex*** |
|  | Semantic verbal fluency | Controlled Oral Word Association Test | ***Frontal Lobe*** |
|  | Category verbal fluency/ Expressive Speech | Animal Naming Test | ***Frontal Lobe*** |
|  | Visual Agnosia | Visual recognition Test | ***Parietal Lobe*** |
|  | Visuo-spatial Ability | RCFT, MAZE | ***Fronto-parital*** |
|  | Visuo-Constructive Ability | RCFT-Copy, Block Designing | ***Parietal Lobe*** |
|  | Visuo-motor coordination | RCFT, MAZE, Coding, Block Designing | ***Parietal Lobe*** |
|  | Visuo-conceptual abilities | Picture Completion | ***Parietal Lobe*** |
