# Supplementary material for: Working Memory Alterations Plays an Essential Role in Developing Global Neuropsychological Impairment in Duchenne Muscular Dystrophy
Source: Front Psychol. 2021 Jan 15;11:613242. doi: 10.3389/fpsyg.2020.613242 (PMC7843380; doi:10.3389/fpsyg.2020.613242)
Supplement: Supplementary file 2 [file Table_2.DOC]

Supplementary Table: 2: Details of various factor indexes obtained from RAVLT and MISIC trials to measure various components of memory functioning

| **S. No** | **Factor index** | **Formula** |
| --- | --- | --- |
| **1** | RAVLT Memory Efficiency Index | {[(delayed recall A/15)/(RAVLT Trials 1–5/75)]+[(delayed recognition hits/15) – (false positive/total number of distracters)]} |
| **2** | Proactive Interference | RAVLT List B/RAVLT trial 1 |
| **3** | Retroactive Interference | RAVLT Immediate Recall/T5 |
| **4** | F Speed | RAVLT Delayed Recall/ RAVLT Immediate Recall |
| **5** | Long Term Percent Retention | DR/T5*100 |
| **6** | Attention Fraction | (DSF-DSB)/ DS |
| **7** | Block Design Efficiency | [(Block design Time/Total time (i.e 450)]*[BD Levels achieved/Total levels (10)] |
